# *N*,*N*′-Bis[(2-methyl­phenyl)sulfon­yl]adipamide

**DOI:** 10.1107/S1600536811007203

**Published:** 2011-03-05

**Authors:** Vinola Z. Rodrigues, Sabine Foro, B. Thimme Gowda

**Affiliations:** a Department of Chemistry, Mangalore University, Mangalagangotri 574 199, Mangalore, India; b Institute of Materials Science, Darmstadt University of Technology, Petersenstrasse 23, D-64287 Darmstadt, Germany

## Abstract

The asymmetric unit of the title compound, C_20_H_24_N_2_O_6_S_2_, comprises one half-mol­ecule, the remaining portion being generated *via* an inversion centre. The dihedral angle between the plane of the benzene ring and the SO_2_—NH—C(O)—C—C segment is 89.9 (1)°. In the crystal, inter­molecular N—H⋯O(S) hydrogen bonds link the mol­ecules into infinite chains in [101].

## Related literature

For related structures, see: Gowda *et al.* (2007 ▶, 2010**a* ▶,b* ▶).
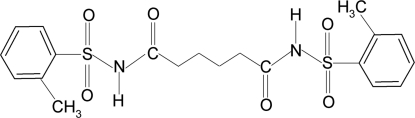


## Experimental

### 

#### Crystal data

C_20_H_24_N_2_O_6_S_2_
*M* *_r_* = 452.53
Monoclinic, 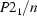

*a* = 11.928 (2) Å
*b* = 5.523 (1) Å
*c* = 16.447 (4) Å
β = 96.05 (2)°
*V* = 1077.5 (4) Å^3^
*Z* = 2
Mo *K*α radiation
μ = 0.29 mm^−1^
*T* = 293 K
0.48 × 0.12 × 0.04 mm

#### Data collection

Oxford Diffraction Xcalibur diffractometer with a Sapphire CCD detector
Absorption correction: multi-scan (*CrysAlis RED*; Oxford Diffraction, 2009 ▶) *T*_min_ = 0.875, *T*_max_ = 0.989
3521 measured reflections
2135 independent reflections
1571 reflections with *I* > 2σ(*I*)
*R*_int_ = 0.037

#### Refinement

*R*[*F* ^2^ > 2σ(*F* ^2^)] = 0.076
*wR*(*F* ^2^) = 0.147
*S* = 1.31
2135 reflections
140 parameters
1 restraint
H atoms treated by a mixture of independent and constrained refinement
Δρ_max_ = 0.52 e Å^−3^
Δρ_min_ = −0.29 e Å^−3^

### 

Data collection: *CrysAlis CCD* (Oxford Diffraction, 2009 ▶); cell refinement: *CrysAlis RED* (Oxford Diffraction, 2009 ▶); data reduction: *CrysAlis RED*; program(s) used to solve structure: *SHELXS97* (Sheldrick, 2008 ▶); program(s) used to refine structure: *SHELXL97* (Sheldrick, 2008 ▶); molecular graphics: *PLATON* (Spek, 2009 ▶); software used to prepare material for publication: *SHELXL97*.

## Supplementary Material

Crystal structure: contains datablocks I, global. DOI: 10.1107/S1600536811007203/vm2082sup1.cif

Structure factors: contains datablocks I. DOI: 10.1107/S1600536811007203/vm2082Isup2.hkl

Additional supplementary materials:  crystallographic information; 3D view; checkCIF report

## Figures and Tables

**Table 1:** Hydrogen-bond geometry (Å, °)

*D*—H⋯*A*	*D*—H	H⋯*A*	*D*⋯*A*	*D*—H⋯*A*
N1—H1*N*⋯O1^i^	0.84 (2)	2.09 (2)	2.917 (4)	166 (4)

Symmetry code: (i) 
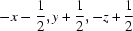
.

**Fig. 1.**
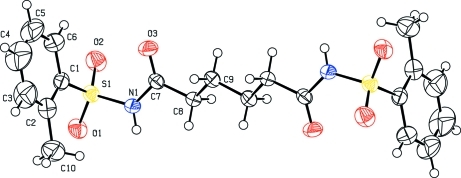
Molecular structure of (I), showing the atom labelling scheme and displacement ellipsoids are drawn at the 50% probability level.

**Fig. 2.**
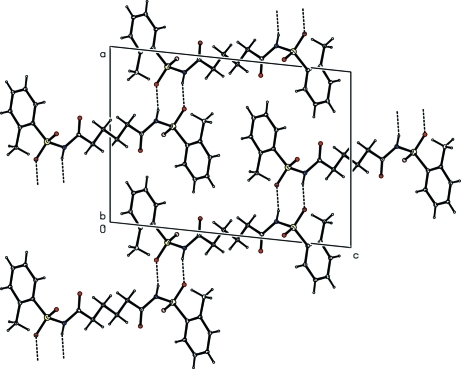
Molecular packing of (I) with hydrogen bonding shown as dashed lines.

## supplementary crystallographic information

### Comment

The sulfonamide moiety is an important constituent of many biologically important compounds. As a part of studying the substituent effects on the structures of this class of compounds (Gowda *et al.*, 2007, 2010**a*,b*), in the present work, the structure of *N*,*N*-bis(2-methylphenylsulfonyl)-adipamide has been determined (Fig.1). The asymmetric unit comprises half of a molecule, the remaining portion being generated via an inversion centre. The conformation of the N—H and C=O bonds in the C—SO_2_—NH—C(O)—C—C segment is *anti* to each other and the amide O atom is also *anti* to the H atoms attached to the adjacent C atom. Further, the conformation of the N—H bond in the amide fragment is *syn* to the *ortho*-methyl group in the adjacent benzene ring. The molecule is bent at the S atom with the C—SO_2_—NH—C(O) torsion angle of -63.7 (4)°. Further, the S1—N1—C7—C8 and C7—N1—S1—O1 segments are nearly linear. The torsion angles C2—C1—S1—N1 and C6—C1—S1—N1 are -71.3 (4)° and 106.9 (4)°, respectively.

The dihedral angle between the planes of the benzene ring and the SO_2_—NH—C(O)—C—C segment is 89.9 (1)°.

N—H···O1(S) H-bond formation results in an S=O1 bond longer than the S=O2 bond. A series of N—H···O(S) intermolecular hydrogen bonds (Table 1) link the molecules into infinite chains running in the [101] direction (Fig. 2).

### Experimental

*N*,*N*-Bis(2-methylphenylsulfonyl)-adipamide was prepared by refluxing a mixture of adipic acid (0.01 mol) with *o*-toluenesulfonamide (0.02 mol) and POCl_3_ for 1 hr on a water bath. The reaction mixture was allowed to cool and added ether to it. The solid product obtained was filtered, washed thoroughly with ether and hot ethanol. The compound was recrystallized to the constant melting point and was characterized by its infrared and NMR spectra.

Needle like colorless single crystals used in the X-ray diffraction studies were grown by a slow evaporation of a solution of the compound in ethanol at room temperature.

### Refinement

The H atom of the NH group was located in a difference map and later restrained to the distance N—H = 0.86 (2) Å. The other H atoms were positioned with idealized geometry using a riding model with ring C—H distance = 0.93 Å, methylene C—H = 0.97 Å  and methyl C—H = 0.96 Å. All H atoms were refined with isotropic displacement parameters (set to 1.2 times of the *U*_eq_ of the parent atom).

### Crystal data

**Table:**

C_20_H_24_N_2_O_6_S_2_	*F*(000) = 476
*M**_r_* = 452.53	*D*_x_ = 1.395 Mg m^−^^3^
Monoclinic, *P*2_1_/*n*	Mo *K*α radiation, λ = 0.71073 Å
Hall symbol: -P 2yn	Cell parameters from 1186 reflections
*a* = 11.928 (2) Å	θ = 2.9–27.9°
*b* = 5.523 (1) Å	µ = 0.29 mm^−^^1^
*c* = 16.447 (4) Å	*T* = 293 K
β = 96.05 (2)°	Needle, colourless
*V* = 1077.5 (4) Å^3^	0.48 × 0.12 × 0.04 mm
*Z* = 2	

### Data collection

**Table:**

Oxford Diffraction Xcalibur diffractometer with a Sapphire CCD detector	2135 independent reflections
Radiation source: fine-focus sealed tube	1571 reflections with *I* > 2σ(*I*)
graphite	*R*_int_ = 0.037
Rotation method data acquisition using ω scans	θ_max_ = 26.4°, θ_min_ = 3.4°
Absorption correction: multi-scan (*CrysAlis RED*; Oxford Diffraction, 2009)	*h* = −14→13
*T*_min_ = 0.875, *T*_max_ = 0.989	*k* = −5→6
3521 measured reflections	*l* = −14→20

### Refinement

**Table:**

Refinement on *F*^2^	Primary atom site location: structure-invariant direct methods
Least-squares matrix: full	Secondary atom site location: difference Fourier map
*R*[*F*^2^ > 2σ(*F*^2^)] = 0.076	Hydrogen site location: inferred from neighbouring sites
*wR*(*F*^2^) = 0.147	H atoms treated by a mixture of independent and constrained refinement
*S* = 1.31	*w* = 1/[σ^2^(*F*_o_^2^) + (0.0079*P*)^2^ + 2.6127*P*] where *P* = (*F*_o_^2^ + 2*F*_c_^2^)/3
2135 reflections	(Δ/σ)_max_ = 0.001
140 parameters	Δρ_max_ = 0.52 e Å^−^^3^
1 restraint	Δρ_min_ = −0.28 e Å^−^^3^

### Special details

**Table:**

Geometry. All e.s.d.'s (except the e.s.d. in the dihedral angle between two l.s. planes) are estimated using the full covariance matrix. The cell e.s.d.'s are taken into account individually in the estimation of e.s.d.'s in distances, angles and torsion angles; correlations between e.s.d.'s in cell parameters are only used when they are defined by crystal symmetry. An approximate (isotropic) treatment of cell e.s.d.'s is used for estimating e.s.d.'s involving l.s. planes.
Refinement. Refinement of *F*^2^ against ALL reflections. The weighted *R*-factor *wR* and goodness of fit *S* are based on *F*^2^, conventional *R*-factors *R* are based on *F*, with *F* set to zero for negative *F*^2^. The threshold expression of *F*^2^ > σ(*F*^2^) is used only for calculating *R*-factors(gt) *etc*. and is not relevant to the choice of reflections for refinement. *R*-factors based on *F*^2^ are statistically about twice as large as those based on *F*, and *R*- factors based on ALL data will be even larger.

### Fractional atomic coordinates and isotropic or equivalent isotropic displacement parameters (Å^2^)

**Table:**

	*x*	*y*	*z*	*U*_iso_*/*U*_eq_	
S1	−0.08049 (9)	−0.2084 (2)	0.23912 (6)	0.0351 (3)	
O1	−0.1885 (2)	−0.2313 (6)	0.19216 (18)	0.0455 (8)	
O2	−0.0301 (3)	−0.4181 (6)	0.27714 (19)	0.0516 (9)	
O3	0.0740 (2)	0.0040 (6)	0.37419 (19)	0.0477 (9)	
N1	−0.1032 (3)	−0.0087 (7)	0.3094 (2)	0.0369 (9)	
H1N	−0.165 (2)	0.064 (7)	0.300 (3)	0.044*	
C1	0.0160 (3)	−0.0737 (8)	0.1790 (3)	0.0369 (10)	
C2	−0.0143 (4)	0.1150 (9)	0.1259 (3)	0.0482 (12)	
C3	0.0713 (5)	0.2137 (13)	0.0847 (4)	0.0775 (19)	
H3	0.0547	0.3411	0.0485	0.093*	
C4	0.1795 (6)	0.1268 (15)	0.0966 (4)	0.092 (2)	
H4	0.2356	0.1986	0.0696	0.110*	
C5	0.2054 (5)	−0.0632 (15)	0.1476 (4)	0.082 (2)	
H5	0.2783	−0.1248	0.1535	0.098*	
C6	0.1249 (4)	−0.1647 (11)	0.1902 (3)	0.0589 (15)	
H6	0.1428	−0.2924	0.2261	0.071*	
C7	−0.0216 (3)	0.0753 (8)	0.3696 (2)	0.0324 (9)	
C8	−0.0659 (3)	0.2492 (8)	0.4283 (2)	0.0345 (10)	
H8A	−0.1013	0.1582	0.4691	0.041*	
H8B	−0.1233	0.3495	0.3989	0.041*	
C9	0.0242 (3)	0.4117 (8)	0.4713 (2)	0.0361 (10)	
H91	0.0808	0.3124	0.5021	0.043*	
H92	0.0608	0.5012	0.4308	0.043*	
C10	−0.1313 (4)	0.2174 (11)	0.1094 (3)	0.0661 (16)	
H10A	−0.1779	0.1063	0.0761	0.079*	
H10B	−0.1282	0.3693	0.0814	0.079*	
H10C	−0.1625	0.2418	0.1603	0.079*	

### Atomic displacement parameters (Å^2^)

**Table:**

	*U*^11^	*U*^22^	*U*^33^	*U*^12^	*U*^13^	*U*^23^
S1	0.0338 (5)	0.0381 (6)	0.0326 (5)	−0.0040 (5)	−0.0005 (4)	−0.0031 (5)
O1	0.0365 (16)	0.055 (2)	0.0431 (17)	−0.0145 (16)	−0.0039 (13)	−0.0087 (16)
O2	0.064 (2)	0.041 (2)	0.049 (2)	0.0036 (17)	0.0004 (16)	0.0035 (17)
O3	0.0293 (16)	0.059 (2)	0.053 (2)	0.0087 (15)	−0.0038 (13)	−0.0119 (17)
N1	0.0268 (18)	0.049 (2)	0.0343 (19)	0.0053 (17)	−0.0021 (15)	−0.0065 (18)
C1	0.031 (2)	0.045 (3)	0.035 (2)	−0.005 (2)	0.0055 (18)	−0.008 (2)
C2	0.055 (3)	0.047 (3)	0.042 (3)	−0.002 (2)	0.006 (2)	−0.002 (2)
C3	0.087 (4)	0.081 (5)	0.068 (4)	−0.008 (4)	0.030 (3)	0.020 (4)
C4	0.069 (4)	0.114 (6)	0.099 (5)	−0.029 (4)	0.044 (4)	0.007 (5)
C5	0.043 (3)	0.125 (6)	0.082 (5)	−0.003 (4)	0.023 (3)	−0.013 (5)
C6	0.041 (3)	0.083 (4)	0.054 (3)	0.007 (3)	0.007 (2)	−0.001 (3)
C7	0.030 (2)	0.037 (2)	0.029 (2)	−0.0020 (19)	−0.0008 (17)	0.0015 (19)
C8	0.029 (2)	0.044 (3)	0.030 (2)	0.0057 (19)	−0.0005 (16)	−0.002 (2)
C9	0.034 (2)	0.041 (3)	0.033 (2)	−0.001 (2)	−0.0007 (17)	−0.004 (2)
C10	0.072 (4)	0.061 (4)	0.065 (4)	0.015 (3)	0.006 (3)	0.016 (3)

### Geometric parameters (Å, °)

**Table:**

S1—O2	1.419 (3)	C4—H4	0.9300
S1—O1	1.436 (3)	C5—C6	1.368 (8)
S1—N1	1.641 (4)	C5—H5	0.9300
S1—C1	1.760 (4)	C6—H6	0.9300
O3—C7	1.201 (5)	C7—C8	1.497 (6)
N1—C7	1.393 (5)	C8—C9	1.516 (6)
N1—H1N	0.843 (19)	C8—H8A	0.9700
C1—C2	1.383 (7)	C8—H8B	0.9700
C1—C6	1.387 (6)	C9—C9^i^	1.514 (8)
C2—C3	1.394 (7)	C9—H91	0.9700
C2—C10	1.504 (7)	C9—H92	0.9700
C3—C4	1.372 (9)	C10—H10A	0.9600
C3—H3	0.9300	C10—H10B	0.9600
C4—C5	1.358 (10)	C10—H10C	0.9600
			
O2—S1—O1	118.7 (2)	C5—C6—C1	118.7 (6)
O2—S1—N1	109.39 (19)	C5—C6—H6	120.6
O1—S1—N1	103.45 (18)	C1—C6—H6	120.6
O2—S1—C1	108.7 (2)	O3—C7—N1	121.6 (4)
O1—S1—C1	109.7 (2)	O3—C7—C8	124.5 (4)
N1—S1—C1	106.1 (2)	N1—C7—C8	113.9 (3)
C7—N1—S1	124.7 (3)	C7—C8—C9	113.6 (3)
C7—N1—H1N	120 (3)	C7—C8—H8A	108.8
S1—N1—H1N	114 (3)	C9—C8—H8A	108.8
C2—C1—C6	122.4 (4)	C7—C8—H8B	108.8
C2—C1—S1	122.1 (3)	C9—C8—H8B	108.8
C6—C1—S1	115.5 (4)	H8A—C8—H8B	107.7
C1—C2—C3	116.5 (5)	C9^i^—C9—C8	112.0 (4)
C1—C2—C10	124.9 (4)	C9^i^—C9—H91	109.2
C3—C2—C10	118.6 (5)	C8—C9—H91	109.2
C4—C3—C2	121.4 (6)	C9^i^—C9—H92	109.2
C4—C3—H3	119.3	C8—C9—H92	109.2
C2—C3—H3	119.3	H91—C9—H92	107.9
C5—C4—C3	120.5 (6)	C2—C10—H10A	109.5
C5—C4—H4	119.8	C2—C10—H10B	109.5
C3—C4—H4	119.8	H10A—C10—H10B	109.5
C4—C5—C6	120.5 (6)	C2—C10—H10C	109.5
C4—C5—H5	119.7	H10A—C10—H10C	109.5
C6—C5—H5	119.7	H10B—C10—H10C	109.5
			
O2—S1—N1—C7	53.4 (4)	C1—C2—C3—C4	0.1 (9)
O1—S1—N1—C7	−179.3 (4)	C10—C2—C3—C4	−179.4 (6)
C1—S1—N1—C7	−63.7 (4)	C2—C3—C4—C5	1.7 (11)
O2—S1—C1—C2	171.1 (4)	C3—C4—C5—C6	−2.5 (11)
O1—S1—C1—C2	39.9 (4)	C4—C5—C6—C1	1.5 (10)
N1—S1—C1—C2	−71.3 (4)	C2—C1—C6—C5	0.3 (8)
O2—S1—C1—C6	−10.7 (4)	S1—C1—C6—C5	−177.8 (5)
O1—S1—C1—C6	−141.9 (4)	S1—N1—C7—O3	−0.2 (6)
N1—S1—C1—C6	106.9 (4)	S1—N1—C7—C8	−177.5 (3)
C6—C1—C2—C3	−1.1 (7)	O3—C7—C8—C9	25.3 (6)
S1—C1—C2—C3	176.9 (4)	N1—C7—C8—C9	−157.5 (4)
C6—C1—C2—C10	178.4 (5)	C7—C8—C9—C9^i^	178.8 (4)
S1—C1—C2—C10	−3.6 (7)		

Symmetry codes: (i) −*x*, −*y*+1, −*z*+1.

### Hydrogen-bond geometry (Å, °)

**Table:**

*D*—H···*A*	*D*—H	H···*A*	*D*···*A*	*D*—H···*A*
N1—H1N···O1^ii^	0.84 (2)	2.09 (2)	2.917 (4)	166 (4)

Symmetry codes: (ii) −*x*−1/2, *y*+1/2, −*z*+1/2.
